# Prevalence and correlates of ‘sexual competence’ at first heterosexual intercourse among young people in Britain

**DOI:** 10.1136/bmjsrh-2018-200160

**Published:** 2019-01-14

**Authors:** Melissa J Palmer, Lynda Clarke, George B Ploubidis, Kaye Wellings

**Affiliations:** 1 Department of Population Health, London School of Hygiene and Tropical Medicine, London, UK; 2 Department of Social Science, UCL Institute of Education, University College London, London, UK; 3 Department of Public Health, Environments and Society, London School of Hygiene and Tropical Medicine, London, UK

**Keywords:** surveys, first sexual intercourse, young people, sexual behaviour

## Abstract

**Background:**

A greater understanding of the circumstances of first sexual intercourse, as opposed to an exclusive focus on age at occurrence, is required in order that sexual health and well-being can be promoted from the onset of sexual activity.

**Methods:**

We used data from the third National Survey of Sexual Attitudes and Lifestyles (Natsal-3) conducted in Britain. Participants were categorised as ‘sexually competent’ at first heterosexual intercourse if the following self-reported criteria applied to the event: contraceptive use, autonomy of decision, both partners ‘equally willing’, and occurrence at the perceived ‘right time’. We examined the prevalence of ‘sexual competence’, and its component parts, by age at first intercourse among 17–24-year-olds. Using multivariable logistic regression, we explored associations between sexual competence and potential explanatory factors.

**Results:**

Variation in ‘sexual competence’ and its component parts was associated with, but not fully explained by, age at first sex: 22.4% and 36.2% of men and women who had first sex at age 13–14 years were categorised as ‘sexually competent’, rising to 63.7% and 60.4% among those aged ≥18 years at first intercourse. Lack of sexual competence was independently associated with: first intercourse before the age of 16 years, area-level deprivation (men only), lower educational level, black ethnicity (women only), reporting ‘friends’ as main source of learning about sex (women only), non-’steady’ relationship at first sex, and uncertainty of first partner’s virginity status.

**Conclusions:**

A substantial proportion of young people in Britain transition into sexual activity under circumstances incompatible with positive sexual health. Social inequalities in sexual health are reflected in the context of first intercourse.

**Key messages**

- A substantial proportion of young people in Britain transition into sexual activity under circumstances that are incompatible with positive sexual health.
- Adverse circumstances of first sexual intercourse were associated with socio-economic status, educational level, source of sex education, relationship with, and virginity status of, the first partner.
- An exclusive focus on chronological age neglects the importance of contextual circumstances in defining the nature of first sexual intercourse.

## Introduction

The context in which first sexual intercourse occurs generally receives less empirical attention than chronological age at first sexual intercourse. However, an exclusive focus on age neglects individual differences in physical, social and psychological maturity, as well as the emphasis placed by young people themselves on the circumstances in which first sex occurred in evaluating their experiences.^1^ As a result, some have argued for a more nuanced concept of readiness and appropriateness of timing of first sexual intercourse.^2^

The concept of ‘sexual competence’ represents an alternative approach to timing of first sexual intercourse, considering the contextual attributes of the event, rather than simply age at occurrence. This departs from the traditional framing of all sexual activity among teenagers as problematic, and recognises that young age alone does not threaten sexual health, any more than older age safeguards it.^3^ With reference to prior work on the role of ‘interactional competence’ in negotiating sexual behaviour,^4 5^ ‘sexual competence’ was operationalised specifically in relation to *first* heterosexual intercourse by Wellings *et al* 6 using four variables measured in the second National Survey of Sexual Attitudes and Lifestyles (Natsal-2). Participants were classified as ‘sexually competent’ at first intercourse if they reported that the event was characterised by contraceptive protection, autonomy of decision, equal willingness of both partners, and that it had occurred at the ‘right time’.

The use of these domains in defining sexual competence may be considered compatible with the definition of sexual health endorsed by the WHO,^7^ highlighting the importance of not only physical health, but also mental and social aspects, referring to a "positive and respectful approach to…sexual relationships" and "safe sexual experiences, free of coercion".

In this study, we examined the prevalence of ‘sexual competence’ and its component variables in a representative population-based sample of 17–24-year-olds living in Britain. Further, we examined the antecedent factors that are associated with a lack of ‘sexual competence’ at first sexual intercourse. The public health relevance of this study is two-fold. First, given its compatibility with the WHO definition of sexual health, the concept of sexual competence is likely to represent first sexual intercourse that is consistent with well-being and health. Second, studies have shown that psychosocial factors relating to first intercourse, such as autonomy and the emotional experience, are associated with sexual health outcomes.^8–10^ We have shown lack of sexual competence at first sex (the term ‘sex’ is used specifically in relation to heterosexual intercourse unless explicitly stated otherwise) to be associated with poor subsequent sexual health, as defined by self-reported sexually transmitted infection (STI) diagnosis, testing positive for human papillomavirus (HPV), lower sexual function, unplanned pregnancy, and experience of non-volitional sex.^11^

## Methods

### Participants

The Third National Survey of Sexual Attitudes and Lifestyles (Natsal-3) is a stratified probability sample survey of 15 162 men and women aged 16–74 years, resident in Britain, conducted in 2010–2012.^12^ We restricted analyses to sexually experienced respondents aged 17–24 years (n=2825) to ensure relevancy to the recent cohort becoming sexually active in Britain. In order to examine the relationship between education and sexual competence, participants aged 16 years at interview were excluded because they could not be ascribed an educational level.

### Measures

Participants were asked about their age at and experience of first heterosexual intercourse in the face-to-face component of the interview. These questions were asked with the use of show cards so that respondents did not have to verbalise any sexually explicit terms, instead quoting the letter that corresponded to their preferred answer option, and to help preserve confidentiality (in case of being overheard by other household members). For participants reporting first sexual intercourse at age 12  years or younger, questions about circumstances were asked about their first experience since turning age 13 years due to ethical concerns relating to probing questions about early non-consensual encounters.^12^ The questions relating to the experience of first intercourse sought to measure whether partners were both equally willing to engage in sexual intercourse; whether the decision to have sex was autonomous (not due to factors external to the self, such as peer pressure or drunkenness); whether the respondent felt their first experience of sexual intercourse had happened at the ‘right’ time; and whether a reliable method of contraception had been used (contraceptive pill or condom) (survey questions have been previously reported).^11^ As in the study by Wellings *et al*,^6^ the measure of sexual competence constructed using these four variables was as follows: respondents who endorsed all four of these items were categorised as ‘sexually competent’ at first intercourse, and respondents who endorsed fewer than all four were categorised as not ‘sexually competent’ at first intercourse. Respondents reporting that their partner was ‘more willing’ at first sex were filtered to an additional question asking whether they were ‘forced’. Those reporting forced first sex were excluded from analyses (n=22) as it was considered inappropriate to classify these respondents in terms of ‘sexual competence’.

Potential explanatory variables were selected with the aim of representing key influences in childhood and adolescence, along with those relating to the more immediate context of first sex. Two indicators relating to socio-economic status were examined: the area-level Index of Multiple Deprivation quintiles,^13^ and educational level of the participant. The ethnicity of participants, and their family structure (whether they lived with both parents) at age 14 years, provided further contextual information. Two variables relating to learning about sexual matters, which potentially have a more direct influence on sexual behaviour, were examined: one based on a question asking participants to identify the ‘main’ source from which they learnt about sexual matters when growing up; the other asked about the level/difficulty of discussing sex with their parents during their teenage years. Finally, factors relating to the immediate context of first sexual intercourse were explored, including the nature of the relationship, the age of the participant at first sex and how this compared with the age of the partner, and the prior sexual experience of the partner.

### Statistical analysis

We present the prevalence of ‘sexual competence’, and the measure’s component parts by age at first sexual intercourse. Unadjusted odds ratios were calculated to examine variation in the prevalence of ‘sexual competence’ at first sex by potential explanatory factors.

Multivariable logistic regression was used to determine which factors were independently associated with ‘sexual competence’ at first sex. Two multivariable regression models are presented. The first model includes the variables relating to socio-demographic background factors, how the respondent learnt about sex, age at first sex. The second model also includes the variables indicative of the relationship context in which first sex occurred. This two-stage approach was employed in order that we could examine the independent associations between sexual competence and the variables conceptualised as more distally related to the outcome, before separately evaluating the variables considered to be more proximal to event, adjusted for those at more distal levels. All analyses were conducted using the Stata (Version 13) survey commands, accounting for the weighting, clustering and stratification of the survey data.

### Patient and public involvement

Patients were not involved in this study.

## Results

### Prevalence and unadjusted odds ratios

Table 1 shows the proportion of 17–24-year-old respondents who reported the following conditions of first intercourse: unequal willingness; a non-autonomous decision; that sex had not happened at the ‘right time’; and non-use of contraception, by gender and age at first sexual intercourse. The most commonly reported negative feature of first sex was that it was not felt to have occurred at the ‘right time’ (39.7% of women and 26.5% of men). Approximately 10% of young people did not use a reliable contraceptive method at first sex. Among women, a general pattern was observed whereby those who were younger at first sex more commonly reported adverse contextual factors, with statistically significant trends observed for perceived timing, equal willingness, and non-autonomous decision-making. Among men, such a trend was observed for perceived timing and contraceptive use. Some 77.7% of women and 64.7% of men who reported first intercourse at age 13–14 years were categorised as not ‘sexually competent’, declining to 36.3% and 39.6% among those aged ≥18 years at first intercourse.

**Table 1:** Proportion of 17–24-year-olds reporting certain circumstances at first sex by age at first sex

Age at first sex (years)	Not equally willing	Not the ’right time'	Non-autonomous reason	Did not use reliable contraception	Not ’sexually competent'	N (unweighted/ weighted)*
	% (95% CI)	% (95% CI)	% (95% CI)	% (95% CI)	% (95% CI)	
Women						
13–14	28.4 (22.7 to 34.9)	71.6 (65.2 to 77.2)	28.8 (23.2 to 35.0)	17.4 (13.0 to 22.8)	77.7 (71.5 to 82.9)	249/119
15	16.0 (12.4 to 20.4)	53.8 (48.1 to 59.5)	23.6 (19.0 to 28.8)	10.2 (7.4 to 13.9)	61.5 (55.7 to 66.9)	352/185
16	15.7 (12.1 to 20.7)	30.8 (26.2 to 35.8)	13.9 (10.8 to 17.8)	6.8 (4.8 to 9.5)	44.9 (39.8 to 50.0)	473/260
17	17.9 (12.4 to 25.0)	31.4 (25.2 to 38.4)	14.3 (10.2 to 19.6)	10.9 (7.2 to 16.2)	47.6 (40.7 to 54.6)	262/152
18–24	13.4 (9.0 to 19.4)	22.1 (16.3 to 29.3)	7.7 (4.4 to 13.1)	11.3 (7.5 to 16.7)	36.3 (29.3 to 44.0)	227/163
P value†	**0.009**	**<0.001**	**<0.001**	**0.234**	**<0.001**	
All	17.4 (15.4 to 19.7)	39.7 (37.0 to 42.4)	17.0 (15.1 to 19.1)	10.5 (8.9 to 12.3)	51.7 (48.9 to 54.5)	
N (unweighted/ weighted)	1567/880	1562/877	1543/865	1566/880	1563/878	
Men						
13–14	16.6 (11.1 to 24.1)	49.0 (41.4 to 56.6)	14.6 (9.5 to 21.6)	25.0 (19.2 to 31.9)	64.7 (57.3 to 71.5)	210/148
15	8.0 (5.0 to 12.4)	29.6 (23.8 to 36.0)	15.3 (11.4 to 20.4)	10.9 (7.4 to 15.8)	47.3 (40.7 to 53.9)	262/186
16	6.7 (4.1 to 10.8)	20.1 (15.1 to 26.2)	11.4 (8.0 to 16.1)	5.7 (3.5 to 8.9)	34.3 (28.5 to 40.6)	318/234
17	13.1 (7.6 to 21.6)	15.6 (10.7 to 22.2)	7.9 (4.7 to 12.8)	9.5 (6.1 to 14.5)	38.0 (30.7 to 46.0)	214/160
18–24	7.4 (4.6 to 11.6)	23.0 (16.8 to 30.6)	12.4 (8.3 to 18.1)	12.8 (8.6 to 18.6)	39.6 (32.3 to 47.3)	217/180
P value†	**0.133**	**<0.001**	**0.161**	**0.014**	**<0.001**	
All	9.8 (7.9 to 12.2)	26.5 (23.8 to 29.5)	12.3 (10.3 to 14.6)	12.0 (10.2 to 14.1)	43.6 (40.4 to 46.9)	
N (unweighted/ weighted)‡	1226/912	1218/906	1221/908	1225/911	1221/908	

*Values of n displayed are the denominators used for calculating the proportions not ‘sexually competent’. Denominators for other components vary slightly due to non-response to individual items.

†P value for test for trend.

‡Denominator for ‘sexual competence’ exceeds that of some components: respondents with missing data on any of the components were classified as not sexually competent if any of the non-missing values indicated unequal willingness, not the right time, non-autonomous reason, or non-use of reliable contraception.

Table 2 shows the prevalence and unadjusted odds ratios of a lack of sexual competence at first sex according to potential explanatory factors. Among both men and women, lack of sexual competence was associated with: living in a more deprived area; lower education level; not living with both parents at age 14 years; first sex occurring before age 16 years; reporting having not been in a ‘steady’ relationship at first sex; the virginity status of the first sexual partner (with the highest odds of lacking sexual competence among those who were uncertain of their partner’s virginity status); and having an older first sexual partner. Among women only, a lack of sexual competence was also associated with black ethnicity; reporting ‘friends’ or ‘other’ as the main source of learning about sexual matters while growing up; and lack of discussion with parents about sexual matters when growing up.

**Table 2:** Proportions and unadjusted odds ratios of respondents aged 17–24 years at interview who were not sexually competent at first sex, by explanatory variables.

Explanatory variables	Men	95% CI	OR	95% CI	P value*	N (unweighted/ weighted)	Women	95% CI	OR	95% CI	P value*	N (unweighted/ weighted)
	%						%					
IMD quintile												
1: Least deprived	27.7	21.5 to 34.8	1		<0.001	216/158	43.9	38.0 to 50.0	1		0.008	263/152
2	38.9	32.3 to 45.9	1.66	1.07 to 2.57		233/177	49.0	42.6 to 55.5	1.23	0.86 to 1.75		280/157
3	40.4	33.7 to 47.6	1.77	1.14 to 2.77		231/164	46.3	39.9 to 52.8	1.10	0.78 to 1.56		283/170
4	52.7	45.2 to 60.0	2.91	1.85 to 4.55		258/213	58.4	52.6 to 64.0	1.79	1.28 to 2.52		339/195
5: Most deprived	53.5	47.0 to 59.9	3.00	1.97 to 4.59		283/197	57.6	51.9 to 63.1	1.73	1.24 to 2.42		398/204
Education level of respondent												
Left school at 17+ years	37.3	33.3 to 41.5	1		<0.001	718/550	46.3	42.8 to 49.8	1		<0.001	930/550
Left school at 16 years with qualifications	51.2	45.6 to 56.6	1.76	1.33 to 2.33		382/262	59.2	54.1 to 64.1	1.68	1.31 to 2.16		479/245
Left school at 16 years with no qualifications	65.4	53.8 to 75.4	3.17	1.89 to 5.30		89/66	70.3	60.0 to 78.8	2.74	1.71 to 4.40		116/55
Ethnic group												
White	42.4	39.1 to 45.8	1		0.143	1094/802	50.1	47.3 to 53.0	1		0.020	1396/768
Mixed	64.6	46.7 to 79.2	2.47	1.16 to 5.26		38/27	64.2	50.8 to 75.7	1.79	1.02 to 3.12		65/36
Asian	48.4	32.5 to 64.6	1.27	0.65 to 2.50		43/40	45.7	29.1 to 63.3	0.84	0.40 to 1.74		38/27
Black	43.8	26.0 to 63.2	1.06	0.47 to 2.36		32/27	76.5	59.2 to 87.9	3.24	1.44 to 7.28		42/33
Chinese and ’other'	57.2	29.3 to 81.2	1.81	0.56 to 5.82		14/13	55.2	31.6 to 76.7	1.23	0.46 to 3.27		20/14
Family structure											<0.001	
Both parents	41.3	37.3 to 45.4	1		0.027	816/643	47.6	44.0 to 51.2	1			928/555
One/neither parent	49.2	43.7 to 54.7	1.38	1.04 to 1.83		405/265	58.6	54.3 to 62.8	1.56	1.25 to 1.96		634/322
Main source of information about sexual matters												
Lessons at school	40.2	34.6 to 46.1	1		0.502	412/310	46.3	41.8 to 50.8	1		0.023	567/322
Mother or father	47.8	36.5 to 59.2	1.36	0.81 to 2.29		92/72	52.0	45.1 to 58.8	1.26	0.90 to 1.75		236/129
Friends	45.5	39.5 to 51.6	1.24	0.89 to 1.73		314/227	55.9	50.7 to 61.1	1.47	1.11 to 1.95		420/230
Other	44.6	39.4 to 50.0	1.20	0.87 to 1.65		396/295	55.5	49.5 to 61.4	1.45	1.07 to 1.95		335/194
Ease discussing sex with parents at age 14 years												
Easy with one/both	42.8	36.2 to 49.6	1		0.300	312/241	46.9	42.1 to 51.8	1		0.051	501/284
Difficult	52.0	40.0 to 63.7	1.45	0.83 to 2.53		87/71	55.3	45.4 to 64.9	1.40	0.90 to 2.20		132/78
Didn’t discuss with either	42.6	38.8 to 46.6	0.99	0.72 to 1.36		759/550	53.8	50.0 to 57.5	1.32	1.03 to 1.68		821/465
Varied depending on topic	31.5	16.9 to 50.9	0.61	0.26 to 1.44		29/25	40.9	28.2 to 54.9	0.78	0.43 to 1.43		59/31
Sex before age 16 years												
No	37.0	32.9 to 41.2	1		<0.001	749/574	43.2	39.6 to 46.8	1		<0.001	962/575
Yes	55.0	49.9 to 60.0	2.08	1.58 to 2.74		472/334	67.8	63.5 to 71.9	2.78	2.17 to 3.55		601/304
Relationship with first sexual partner												
Steady relationship	34.2	30.0 to 38.7	1		<0.001	574/436	39.8	36.3 to 43.3	1		<0.001	968/545
Just/recently met for first time	61.3	53.5 to 68.5	3.04	2.12 to 4.37		207/152	77.3	67.5 to 84.8	5.15	3.07 to 8.64		129/78
Known each other a while, not in steady relationship	47.8	42.4 to 53.3	1.76	1.32 to 2.36		394/286	73.4	68.2 to 78.1	4.19	3.12 to 5.63		384/207
Used to be in steady relationship	52.2	35.9 to 68.1	2.10	1.06 to 4.18		44/33	64.9	48.8 to 78.2	2.80	1.42 to 5.54		61/34.61
Married/living together	–	–	–	–	–	2/1	16.2	4.9, 42.5	0.29	0.08 to 1.13		20/13
Partner’s first time too												
Yes, partner’s first time	37.1	32.9 to 41.5	1		<0.001	577/434	41.6	37.2 to 46.0	1		<0.001	573/318
Think it was first time	52.8	37.1 to 68.0	1.90	0.97 to 3.70		50/43	72.1	55.4 to 84.3	3.63	1.71 to 7.72		43/24
Think it was not first time	59.5	47.8 to 70.2	2.49	1.51 to 4.12		94/69	66.9	55.2 to 76.9	2.85	1.70 to 4.78		82/49
No, not first time	46.0	40.7 to 51.3	1.44	1.09 to 1.91		444/325	55.9	52.1 to 59.7	1.79	1.41 to 2.27		825/466
Age difference between respondent and first sex partner												
Same age	40.2	36.0 to 44.5	1		<0.001	718/535	45.6	41.0 to 50.2	1		0.005	551/314
Partner younger than respondent	38.6	31.3 to 46.5	0.94	0.64 to 1.36		211/168	56.6	41.6 to 70.4	1.56	0.83 to 2.92		54/31
Respondent younger than partner	56.4	49.8 to 62.8	1.92	1.41 to 2.63		286/203	55.0	51.5 to 58.5	1.46	1.16 to 1.85		956/532

*Wald test.

CI, confidence interval; IMD, Index of Multiple Deprivation; OR, odds ratio.

### Multivariable regression analyses

The results of multivariable logistic regression analyses are presented in tables 3 and 4. The first model (Model 1) includes the variables relating to socio-demographic background factors, how the respondent learnt about sex, and age at first sex. In these adjusted models, the majority of associations observed in the crude analyses were retained, although somewhat attenuated. (Model 1, tables 3 and 4).

**Table 3:** Logistic regression examining predictors of lack of sexual competence at first sex, results adjusted for all other variables in table column (Men)

Outcome: not sexually competent at first intercourse	Model 1			Model 2		
	AOR	95% CI	P value	AOR	95% CI	P value
IMD quintile						
1: Least deprived	1		0.003	1		0.006
2	1.58	1.00 to 2.48		1.49	0.95 to 2.34	
3	1.64	1.03 to 2.62		1.55	0.97 to 2.47	
4	2.43	1.51 to 3.92		2.29	1.44 to 3.63	
5: Most deprived	2.21	1.40 to 3.48		2.03	1.28 to 3.22	
Education level of respondent						
Studying for/attained further academic qualifications	1		0.002	1		0.047
No academic qualifications	2.36	1.32 to 4.22		2.27	1.25 to 4.13	
Academic qualifications typically gained at age 16 years	1.54	1.12 to 2.12		1.49	1.08 to 2.05	
Ethnic group						
White	1		0.136	1		0.130
Mixed	2.84	1.18 to 6.84		2.88	1.12 to 7.41	
Asian	1.49	0.73 to 3.05		1.71	0.82 to 3.58	
Black	0.75	0.31 to 1.82		0.75	0.30 to 1.87	
Chinese and ’other'	0.84	0.13 to 5.46		0.85	0.10 to 7.24	
Family structure						
Both parents	1		0.957	1		
One/neither parent	0.99	0.72 to 1.36		1.01	0.74 to 1.39	0.935
Main source of information about sexual matters						
Lessons at school	1		0.646	1		0.754
Mother or father	1.29	0.69 to 2.42		1.22	0.67 to 2.22	
Friends	1.00	0.70 to 1.43		0.95	0.66 to 1.37	
Other	0.88	0.62 to 1.25		0.89	0.62 to 1.27	
Ease discussing sex with parents at age 14 years						
Easy with one/both	1		0.272	1		0.197
Difficult	1.65	0.87 to 3.13		1.66	0.87 to 3.17	
Didn’t discuss with either	0.99	0.69 to 1.43		0.96	0.67 to 1.38	
Varied depending on topic	0.72	0.31 to 1.70		0.64	0.28 to 1.46	
Sex before age 16						
No	1		<0.001	1		0.001
Yes	1.72	1.27 to 2.34		1.71	1.24 to 2.36	
Relationship with first sexual partner						
Steady relationship				1		<0.001
Just/recently met for first time				2.42	1.57 to 3.73	
Known each other a while to not in steady relationship				1.15	0.81 to 1.62	
Used to be in steady relationship				1.56	0.66 to 3.70	
Married/living together				–	–	–
Partner’s first time too						
Yes to partner’s first time				1		0.0639
Think it was first time				1.75	0.87 to 3.53	
Think it was not first time				2.12	1.15 to 3.91	
No to not first time				1.19	0.84 to 1.67	
Age difference between respondent and first sex partner						
Same age				1		0.4316
Partner younger than respondent				0.94	0.63 to 1.41	
Respondent younger than partner				1.25	0.86 to 1.80	
N unweighted			1092			1092
N weighted			817			817

AOR, adjusted odds ratio; CI, confidence interval; IMD, Index of Multiple Deprivation.

**Table 4:** Logistic regression examining predictors of lack of sexual competence at first sex, results adjusted for all other variables in table column (Women)

Outcome: not sexually competent at first intercourse	AOR	Model 1	P value	AOR	Model 2	P value
		95% CI			95% CI	
IMD quintile						
1: Least deprived	1		0.233	1		0.291
2	1.19	0.81 to 1.74		1.18	0.78 to 1.78	
3	0.99	0.68 to 1.43		0.96	0.64 to 1.44	
4	1.44	0.99 to 2.07		1.43	0.97 to 2.12	
5: Most deprived	1.26	0.87 to 1.82		1.22	0.83 to 1.81	
Education level of respondent						
Studying for/attained further academic qualifications	1		0.020	1		0.067
Academic qualifications typically gained at 16 years	1.34	1.02 to 1.76		1.22	0.91 to 1.63	
No academic qualifications	1.81	1.07 to 3.08		1.87	1.06 to 3.32	
Ethnic group						
White	1		0.050	1		0.009
Mixed	1.75	0.87 to 3.52		1.47	0.74 to 2.93	
Asian	1.54	0.67 to 3.52		2.85	1.10 to 7.41	
Black	3.22	1.26 to 8.22		4.67	1.62 to 13.45	
Chinese and ’other'	1.66	0.60 to 4.58		2.97	0.80 to 11.01	
Family structure						
Both parents	1		0.029	1		0.245
One/neither parent	1.34	1.03 to 1.73		1.18	0.89 to 1.56	
Main source of information about sexual matters						
Lessons at school	1		0.071	1		0.536
Mother or father	1.38	0.92 to 2.09		1.33	0.85 to 2.09	
Friends	1.48	1.09 to 2.02		1.20	0.86 to 1.67	
Other	1.25	0.90 to 1.72		1.15	0.80 to 1.64	
Ease discussing sex with parents at age 14 years						
Easy with one/both	1		0.096	1		0.137
Difficult	1.58	0.93 to 2.67		1.51	0.90 to 2.54	
Didn’t discuss with either	1.39	1.02 to 1.90		1.39	0.98 to 1.95	
Varied depending on topic	0.94	0.50 to 1.77		0.91	0.47 to 1.76	
Sex before age 16 years						
No	1		<0.001	1		<0.001
Yes	2.60	2.00 to 3.38		2.93	2.20 to 3.90	
Relationship with first sexual partner						
Steady relationship				1		<0.001
Just/recently met for first time				4.84	2.71 to 8.64	
Known each other a while to not in steady relationship				3.95	2.81 to 5.56	
Used to be in steady relationship				2.95	1.45 to 6.01	
Married/living together				0.14	0.02 to 1.21	
Partner’s first time too						
Yes to partner’s first time				1		<0.001
Think it was first time				3.90	1.62 to 9.36	
Think it was not first time				2.29	1.21 to 4.34	
No to not first time				1.52	1.14 to 2.04	
Age difference between respondent and first sex partner						
Same age				1		0.639
Partner younger than respondent				1.40	0.70 to 2.81	
Respondent younger than partner				1.04	0.79 to 1.37	
N unweighted			1432			1432
N weighted			805			805

AOR, adjusted odds ratio; CI, confidence interval; IMD, Index of Multiple Deprivation.

After adjustment for variables relating to the immediate relational context in which first intercourse occurred (Model 2, table 4), source of learning about sexual matters and communication with parents about sexual matters were no longer associated with sexual competence among women, potentially indicating a mediatory role of these more proximal factors. Lower educational level, black ethnicity, and sex before 16 years retained their associations with a lack of sexual competence at first sex among women, although at a borderline level for the former. Among men, the associations between a sexual competence and IMD quintile, educational level, and sex before 16 retained statistical significance, even after adjustment for the variables relating to the immediate relational context of first sex (Model 2, table 3).

After adjustment for all other variables in the model, the status of the relationship with the first sexual partner retained its strong associations with sexual competence (Model 2, tables 2 and 3). Among men, this association seemed primarily driven by the increased odds of a lack of sexual competence among those reporting they ‘had just/recently met’ their partner compared with those in a ‘steady relationship’ at the time. Respondents’ knowledge of their first sexual partner’s virginity status also continued to be associated with sexual competence among women after adjustment. A similar association, of borderline statistical significance, was also evident among men. Finally, having had an older partner at first sex was no longer associated with lacking sexual competence among men or women in these fully adjusted models.

## Discussion

This study describes the circumstances of first sexual intercourse using a representative population-based sample of young people living in Britain, and provides an exploration of the antecedent factors associated with a novel measure of the first sexual intercourse experience: ‘sexual competence’.

Adverse circumstances of first sex were reported by a substantial proportion of young people. More than a third of women and a quarter of men did not consider that their experience of first sexual intercourse occurred at the ‘right time’, while almost 1 in 5 women reported that they and their partner were not equally willing to have sex on that first occurrence, and a similar proportion of women reported a non-autonomous reason for first sex. While the majority of young people used a reliable contraceptive method at first sex, 1 in 10 did not.

In relation to the composite measure of ‘sexual competence’, over half of women and more than a third of men were categorised as not being ‘sexually competent’ at first sex. Although age at first intercourse was associated with sexual competence, it did not explain all of the variability in sexual competence – at no age did the prevalence of sexual competence approach zero or 100%. This finding supports the proposition that chronological age may be an overly simplistic indicator of the nature of first intercourse. Furthermore, the associations between several antecedent factors and sexual competence at first sex were retained when adjusting for age at first sex. This provides further evidence that the measure of sexual competence represents a distinct dimension of the experience of first intercourse, which is not simply a function of age.

In line with previous research, the stability of the partnership was associated with a more positive first sexual experience.^14–17^ Uncertainty relating to the virginity status of the partner was associated with a lack of sexual competence, potentially suggesting this variable is acting as a proxy for communication between partners. The association between indicators of socio-economic status and sexual competence is consistent with previous research^6 15 18 19^ and may be explained by the effect of limited life aspirations on sexual behaviour.^18 20^ Previous research has found that individuals of lower socio-economic status have lower levels of perceived control,^21 22^ which could be an important psycho-social determinant of sexual competence. The association between ethnicity and sexual competence is consistent with previous research reporting variations in sexual behaviour across different ethnic groups, as is the finding that the association remains after adjustment for broader risk factors (eg, indicators of socio-economic status).^23^ Further research is warranted to examine the drivers of ethnic variations in sexual behaviour and health.

That young women who had discussed sexual matters with their parents, and those who reported school to be their main source from which they learnt about sexual matters, were more likely to have been sexually competent at first sex resonates with previous research.^6 24^ Parental communication, and school-based relationships and sex education, may provide the knowledge and skills required to negotiate a positive and safe sexual experience. However, these associations were not observed among men, even in unadjusted analyses. A possible interpretation is that communication and negotiation skills are less important for men in achieving a first sexual intercourse that they reflect positively on. Prior research reports that men generally give more positive accounts of first intercourse as they are more likely to just be happy that they had sex^25 26^ and less likely to report experiencing pressure from their partner.^25 27^

### Limitations

The response rate to Natsal-3, at 57.7%,^12^ potentially limits representativeness of the findings. Our reliance on observational data means that the associations detected may be due to unmeasured and/or unknown confounders. The Natsal survey relies on retrospective self-reports relating to an event that could have occurred up to a decade earlier; therefore, it is important to consider the potential for recall bias when interpreting the results. This could explain the strong association observed between relationship with partner at first sex and sexual competence at first sex; perhaps those who reflect on the first sexual intercourse positively, and therefore will be classified at sexually competent, will also be more likely to recall that they were in a ‘steady’ relationship at the time. Finally, despite Natsal-3’s large sample size, a relatively small proportion of participants were of non-white British ethnicity (reflecting the ethnic composition of Britain), meaning that analyses involving specific ethnic groups were limited by small numbers.

## Conclusions and implications

A substantial proportion of young people in Britain become sexually active under circumstances that are arguably incompatible with sexual health defined in its broad sense encompassing both physical and psycho-social well-being.

The antecedent factors associated with sexual competence are of public health relevance for understanding where interventions to improve the conditions of first sex may be best targeted. Inequalities in sexual health have commonly been described in terms of the unequal distributions of STIs,^28^ unplanned pregnancies,^29^ and ‘early’ transitions into sexual activity,^30^ across socio-economic groups. The current findings suggest that these inequalities are also reflected in the nature of first intercourse, indicating that greater efforts are required to reduce the disparities that exist from the very onset of sexual activity. While the results indicate that communication with parents about sex and school-based sex education may help towards the achievement of sexual competence among young women, the same cannot be said for men, suggesting that greater consideration needs to be given to how men can best be equipped to have a safe and positive transition into sexual activity.

Previous research has suggested that the experience of first sexual intercourse can have implications for sexual health status later in life.^8–10^ Analyses of Natsal-3 data have identified lack of sexual competence at first intercourse to be a risk factor for poor subsequent sexual health among young people, independently of age at first sex.^11^ Therefore, it is possible that targeted interventions aimed at enabling at-risk young people to have a more positive and healthy first sexual experience may result in improvements in sexual health that continue into adulthood.

As a research tool, the measure of sexual competence was constructed rather opportunistically by Natsal-2 researchers combining existing variables considered to be necessary for a healthy first sexual intercourse. The finding that age at first sex does not explain all of the variation observed in sexual competence, and nor does it account for the associations found with other antecedent factors, suggests that this measure is not merely a function of age at first sex, but rather tapping into a distinct dimension of the experience in itself. These conclusions support the future use of this measure in research concerned with sexual behaviour among young people, and may represent an alternative indicator by which the effectiveness of interventions to improve sexual health can be assessed.
